# Gut–CNS-Axis as Possibility to Modulate Inflammatory Disease Activity—Implications for Multiple Sclerosis

**DOI:** 10.3390/ijms18071526

**Published:** 2017-07-14

**Authors:** Ann-Katrin Fleck, Detlef Schuppan, Heinz Wiendl, Luisa Klotz

**Affiliations:** 1 Department of Neurology, University Hospital Muenster, 48149 Muenster, Germany; ann-katrin.fleck@ukmuenster.de (A.-K.F.); heinz.wiendl@ukmuenster.de (H.W.); 2 Institute of Translational Immunology, University Medical Center of the Johannes Gutenberg University Mainz, 55131 Mainz, Germany; detlef.schuppan@unimedizin-mainz.de; 3 Division of Gastroenterology, Beth Israel Deaconess Medical Center, Harvard Medical School, Boston, MA 02215, USA

**Keywords:** gut–CNS-axis, microbiota, immune system, multiple sclerosis, nutrition

## Abstract

In the last decade the role of environmental factors as modulators of disease activity and progression has received increasing attention. In contrast to classical environmental modulators such as exposure to sun-light or fine dust pollution, nutrition is an ideal tool for a personalized human intervention. Various studies demonstrate a key role of dietary factors in autoimmune diseases including Inflammatory Bowel Disease (IBD), rheumatoid arthritis or inflammatory central nervous system (CNS) diseases such as Multiple Sclerosis (MS). In this review we discuss the connection between diet and inflammatory processes via the gut–CNS-axis. This axis describes a bi-directional communication system and comprises neuronal signaling, neuroendocrine pathways and modulation of immune responses. Therefore, the gut–CNS-axis represents an emerging target to modify CNS inflammatory activity ultimately opening new avenues for complementary and adjunctive treatment of autoimmune diseases such as MS.

## 1. Introduction

The important role of environmental factors in inflammatory disease etiology and pathogenesis has been increasingly studied during recent years. Despite a clear contribution of genetic background and epigenetic modifications, environmental factors have been identified to modulate the susceptibility of immune-driven diseases. In this regard, growing attention has been paid to the impact of nutritional factors for disease pathophysiology. This aspect is particularly interesting, as dietary factors can be easily modified and customized to individual requirements, potentially serving as a complementary approach to accompany conventional treatment strategies [1]. In line with this concept, malnutrition has been associated with chronic inflammatory diseases, such as atherosclerosis, obesity, diabetes mellitus and multiple sclerosis (MS) [1].

MS is a neurodegenerative and demyelinating disease of the central nervous system with a prominent autoimmune component. In particular, young adults at the age between 20 and 40 are affected by this neurological disorder, which is pathophysiologically characterized by perivascular inflammation, the loss of blood brain barrier integrity, disruption of the myelin layer, axonal damage and progressive neuronal degeneration [2,3]. Sensibility disorders, muscle spasms, limb weakness or visual as well as sensory disturbances are characteristic symptoms for neurological disability in MS. So far, MS etiopathogenesis cannot be explained by the genetic background alone. Thus, although epidemiological studies revealed an association between MS and several immune related genes, the concordance rate of 15–30% in monozygotic twins indicates a multi-factorial nature of MS [4,5,6]. Several preclinical studies in MS patients suggested modulating influences of dietary salts [7,8,9], vitamin D supplementation [10,11], extracts of green tea [12] and polyunsaturated fatty acids [13,14,15]. In this line, high concentrations of fat [16,17] and salt [7,8,9], which are key features of the hyper-caloric Western diet [18], promote pro-inflammatory responses of effector T cells and macrophages resulting in an exacerbation of inflammatory processes [16]. Various clinical studies had been performed to examine the emerging potential of dietary factors to influence MS susceptibility. However, a general problem in these studies was the chosen study designs. Thus, a small study size, insufficient inclusion criteria and the absence of adequate controls have limited the validity of these studies [19]. Furthermore, the myriads of interfering co-factors, such as the nutrition-depended modulation of overlapping comorbidities, have to be taken into consideration. Therefore, due to the heterogeneity of study designs, an appropriate comparison of the currently published studies is impossible.

Among some direct effects on host processes, such as the modulation of T cells responses by a Western-style nutrition [18], there is growing evidence that nutritional habits impact the composition as well as the functionality of the gastro-intestinal microbiota [20]. The microbiota of the gut comprises several trillions of microbes, thereby outnumbering the amount of host cells by a factor of 100 [21]. During infancy the core gastro-intestinal microbial composition is determined, in part depending on the mode of delivery at birth and with the intensity of breast feeding [22,23]. Microbial disturbances in these early phases of development via antibiotic treatment, certain infections and also unhealthy nutritional habits, bear the potential to increase susceptibility to future diseases [23,24]. One to three years after birth the adult-like microbiome is established, but continuously underlies dynamic modification by dietary habits [23,24]. Although the uptake of specific nutrients is assumed to mitigate former negative impacts, recent consumption of detrimental dietary factors might deteriorate the status of health [23,24]. Some main functions of the intestinal microbiota are the metabolism of nutrients (such as digestion and fermentation of carbohydrates), the production of bioactive molecules (such as neurotransmitters and vitamins) and the competition with colonizing pathogens [25]. Further, the microbiota is essential for priming the gastro-intestinal immune system to evoke specific immune responses. De Filippo et al. [26] described beneficial effects in the protection against inflammation of African children with traditional dietary habits, who have an altered intestinal microbiota structure compared to European children who consume a modern Western diet. Due to these fundamental functions, the microbiota is an important regulatory counterpart to the host metabolism and immune surveillance. Depending on composition and diversity, the intestinal microbiota can modulate host mechanisms via several pathways, including target cells and structures in immediate proximity to the microbiota, such as the intestinal epithelial cells, immune cells in the gut-associated lymphatic tissue (GALT) and the enteric nervous system (ENS), but also remote structures, such as the liver, the adrenal or pituitary glands and the central nervous system (CNS). The microbiome thereby influences (a) local physiological processes, such as intestinal motility [27], permeability [28] or hormone secretion [29,30]; (b) the functionality of remote organs; or (c) systemic inflammatory processes. More and more attention is being paid to recent findings that the gastro-intestinal microbiota is associated with CNS homeostasis and development [31], but also with neuroimmunological diseases (e.g., MS or Neuromyelitis optica (NMO) [32]), and neuropsychiatric disorders, such as depression [33], schizophrenia [34], autism [35], Parkinson’s disease [36] and Alzheimer’s disease [37,38]. The responsible highly complex network of these interactions is summarized in the gut–CNS-axis, which comprises neuronal connections, neuroendocrine and general humoral pathways, and the immune system. This bi-directional communication system allows modulation of CNS activities by the gut and vice versa, manipulation of the gastro-intestinal tract functionality by the CNS, which is mediated by the enormous innervation rate and high numbers of immune cells in the gut [39]. Therefore, the gut–CNS-axis represents an attractive target to modulate physiological as well as pathological processes in the CNS by nutritional factors.

## 2. Components of the Gut–CNS-Axis

**Figure 1:**
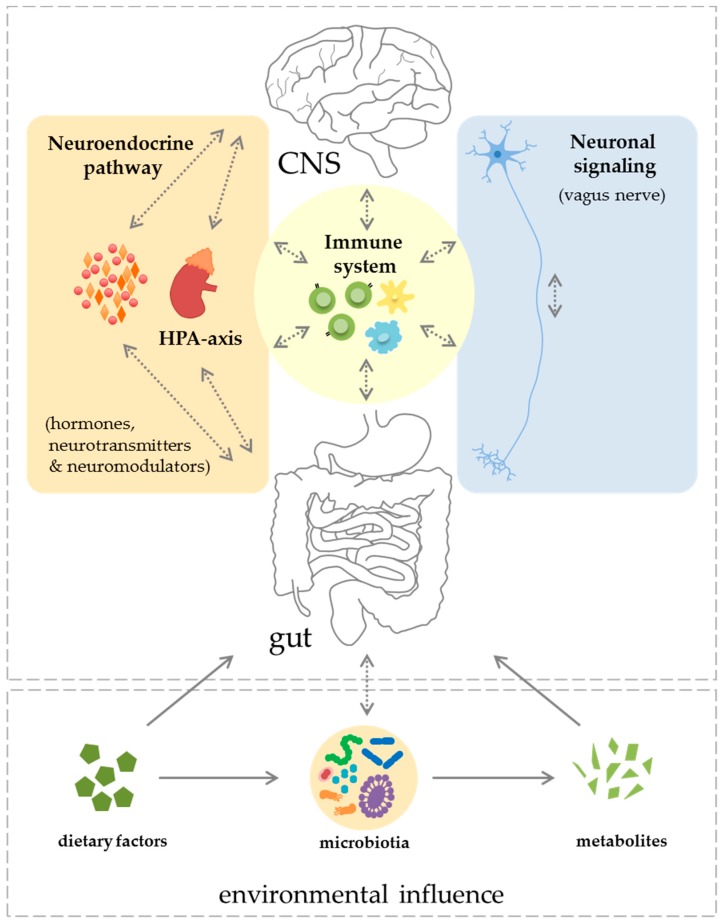
The gut–CNS-axis comprises several mutually interacting systems and signaling pathways. The main components are the neuroendocrine system including the HPA-axis, neuronal signaling via the vagus nerve and the immune system. CNS = central nervous system; HPA-axis = hypothalamic–pituitary–adrenal gland. (⟶ = unidirectional and <⋯> = bidirectional communication).

To further clarify the mutual relationship between the gastro-intestinal tract and the CNS, the individual components are described in detail in the following section and summarized in Figure 1.

### 2.1. Neuronal Signaling and Neuroendocrine Component

The CNS can directly interfere with the gut via sympathetic or parasympathetic branches of the autonomic nerve system (ANS), especially the vagus nerve. Hence, the microbiome can be modulated either directly by bioactive molecules released from the ENS or indirectly via other modes of modification of the microbial environment, such as gastro-intestinal motility, permeability, pH value or mucus secretion [40]. These regulations are mostly mediated by ANS secretion of acetylcholine or catecholamines, which influences ENS circuits [40,41]. Acetylcholine release by the vagus nerve is suggested to suppress secretion of pro-inflammatory tumor necrosis factor alpha (TNFα), interleukin 6 (IL-6) or IL-18. Experiments with vagotomized mice illustrated the critical role of the vagus nerve in the cross-talk between the gastrointestinal tract and the CNS. Treatment of mice with *Lactobacillus rhamnosus* reduced γ-aminobutyric acid (GABA) receptor expression in the brain and thereby induced anxiolytic and anti-depressive effects, which were abrogated in mice after vagotomy [42]. Similary, there was no anxiolytic and behavioral influence of *Bifidobacterium longum* in vagotomized mice with chronic colitis, while an attenuation of psychological comorbidities of colitis was observed after administration of *Bifidobacterium longum* in mice with an intact vagus nerve signaling [43]. On the other hand, the beneficial psychological effect of probiotics may not only be mediated via the vagus nerve, since treatment with *Lactobacillus rhamnosus* and *Bifidobacterium infantis* improve colitis both in sham-operated and vagotomized mice with chronic colitis has been observed [44]. Thus, additional investigations are required to determine strain-specific effects in distinct inflammatory disorders, but also to illuminate other potential mechanisms of action [45].

Another key part of the gut–CNS-axis is the neuroendocrine signaling, which mediates its effects via neurotransmitter release or the hypothalamic–pituitary–adrenal (HPA) axis. Beside the CNS, the intestinal microbiota produces neurotransmitters or neuromodulators and therefore exhibit the potential to directly modulate CNS activities [46]. For instance, over 95% of the endogenous serotonin originates from the gut [30,47]. However, there are also microbiota producing acetylcholine [41,48], tryptamine [49], catecholamines [50] and GABA [42,51]. Furthermore, microbial metabolites can induce secretion of neuromodulatory substances by epithelial enterochromaffine cells, neurons or immune cells. The bacterial metabolites propionic acid, butyric acid and acetic acid are short-chain fatty acids (SCFA) [52], that exert neuromodulatory functions. Indeed, butyric acid exhibits anti-inflammatory and neuroprotective properties via inhibition of histone deacetylases [53,54] and associated epigenetic modulation [55]. This interference in the neuronal communication via neuroendocrine secretion may have a key impact on CNS processes and vice versa, modulating the colonization of intestinal bacteria, resulting in an altered microbiome functionality [56].

The hypothalamic-pituitary-adrenal (HPA) axis comprises the hypothalamus, the pituitary as well as the adrenal gland. In response to stress or specific neuronal inputs (limbic, afferent sympathetic and parasympathetic circuits) the HPA-axis finally releases glucocorticoids (e.g., cortisol in human or corticosterone in rodents), mineralocorticoides or catecholamines, which can alter microbiota composition [56], permeability of the gut epithelium [57], metabolic processes but also immune responses [58,59]. Enhanced levels of corticosterone in stressed mice is associated with intestinal dysbiosis, which is characterized by an increase in the relative abundance of the genus *Clostridium* and a decrease in the relative abundance of the genus *Bacteroides* [60]. Moreover, glucocorticoids are potent immunomodulators with both pro- and anti-inflammatory effects on peripheral and CNS-resident immune cells, depending on the context (reviewed in detail by [59,61]). This could be one explanation why an impaired HPA axis functionality is often associated with inflammatory and autoimmune diseases, such as rheumatoid arthritis, inflammatory bowel disease (IBD) and MS [62]. Thus, the HPA-axis is a powerful system to modulate gut functionality and immune responses, and probiotics can influence the HPA-axis and alter CNS signaling. Probiotic *Lactobacillus* species are known to reduce stress-related HPA-axis responses and elevated glucocoticoid levels, which result in attenuation of stress-related neuroinflammation [57,63].

### 2.2. Immune System

In addition to neuroendocrine signaling, the gastro-intestinal microbiome regulates the development of the host immune system and contributes to an orchestrated immune response. Immune cells are specialized for the recognition of microbial structures or tissue damage, with the need to differentiate between friend and foe. Since these cells reach almost every body tissue and possess the potential to specifically modulate inflammatory processes, the immune system is another powerful component of the gut–CNS-axis.

The presence of microbes is essential for the generation of an efficient host immunity, because they are e.g., required for the formation of GALTs in the gastro-intestinal tract [64]. These immunological structures allow priming of lymphocytes via antigen-presentation and thereby establish a discriminative immune system, which can elicit either defense and inflammation or tolerance, depending on the presented antigen [21,64,65]. Development of the GALT is impaired in germ-free mice. These mice are born and maintained under sterile conditions, thus lacking microbial colonization. Hence, germ-free mice have low levels of lymphocytes and immunoglobulins, which results in a severely comprised innate and adoptive immune system [21,64,65]. Subsequent bacterial colonization of germ-free mice shapes the small intestine morphology and enhances vascularisation, which in turn allows an intense recruitment and activation of immune cells but also activates the previously dormant intestinal immune system in an “uneducated way”, e.g., without the ability to differentiate between harmless and harmful microbiota and nutrients [66]. Hence, microbe-signaling can provoke vigorous cytokine responses, activation of innate immunity and the complement cascade, leading to an alteration of host gene expression (e.g., variation of PPARγ-mediated processes) and an up-regulation of innate as well as adaptive inflammatory responses [66,67]. Microbial-associated molecular patterns (MAMPs) are bacterial components, which are pivotal to the early regulation of inflammatory responses, e.g., via induction of toll-like receptors (TLRs) signaling [68]. Low concentrations of circulating commensal MAMPs (e.g., lipopolysaccharides, peptidoglycans or flagellin) are required for the suppression of inflammatory responses against commensal bacteria and the selective clearance of pathogens [69,70]. Chronic microbe translocation increases the circulating MAMP level, which modifies the secretion profile of various TLR-expressing cells, such as peripheral and tissue-resident immune cells but also CNS-resident glial cells and neurons. Thus results in chronic systemic inflammation and also inflammation of the CNS [68,69,70,71,72]. On the other hand, the capsular polysaccharide A (PSA) is a TLR2-ligand that exhibits specific anti-inflammatory properties. Oral treatment with capsular PSA derived from human commensal bacteria suppresses neuroinflammatory activity in mice with experimental autoimmune encephalomyelitis (EAE) or experimental colitis via enhancement of regulatory T (Treg) cell migratory capacity [73,74,75,76,77]. Further, microbial structures influence brain-resident glial cells, such as yolk sac-derived microglia and astrocytes. Both cell types play a pivotal role in modulation of inflammatory processes [78]. More specifically, germ-free or antibiotic-treated mice show an impaired maturation and functionality of microglia, which could be restored by bacterial colonization or administration of SCFA [79]. Moreover, the anti-inflammatory activities of astrocytes could be induced by certain tryptophan metabolites via upregulation of the aryl hydrocarbon receptor [80]. Beside the maintenance of host homeostasis via modulation of physiological, as well as pathological processes and further the defense against microbes, the hormone- and stress-mediated neuroendocrine pathways just as neuronal signaling interferes with the function of the immune system. Consequently, the immune system decisively modulates other components of the gut–CNS-axis and its functionality.

#### 2.2.1. Innate Immune System

##### Myeloid Cells

The lamina propria of the gastro-intestinal tract accommodates a spectrum of myeloid cells, mainly dendritic cells or macrophages, which play a key role in “first-line” host defense and the modulation of adaptive immunity [81]. The mononuclear phagocytes tolerate commensal bacteria, but upon invasion of pathogenic microbes rapidly increase their antimicrobial activity via secretion of pro-inflammatory mediators and the expression of co-stimulatory molecules [82,83]. Thus, it has been demonstrated that mucosal myeloid cells in the lamina propria surrender their hyporesponsive default state after bacterial invasion, e.g., by dramatically enhancing the production of pro-IL-1β and its processing to bioactive IL-1β by caspase-1 [82]. Therefore, myeloid cells “have learned” to discriminate between pathogenic and protective bacteria and dietary components [82]. Moreover, the rise of antimicrobial activity and the increased expression of co-stimulatory molecules in response to microbial infection are essential for the priming of other immune cells and coordination of the overall host immune response [83]. Compared to mice with normal commensal microbiota, mononuclear phagocytes of germ-free mice are impaired in their antimicrobial activity, in particular in the Type 1-interferon (IFN) response, after microbial infection. Type 1-IFN signaling is required for priming of cytotoxic lymphocytes such as natural killer (NK) T cells [83].

##### Natural Killer T Cells

NK T cells belong to the innate immune cells, since they exhibit the potential to rapidly secrete cytokines in response to infection, but in contrast to other innate population they express also (invariant) T cell receptors. There is evidence that in germ-free mice NK T cells accumulate in the mucosal layers of lung and intestine mediated by the secretion of chemokine CXCL16 by epithelial cells. These NK T cells secrete several potent pro-inflammatory cytokines, such as IL-4, IL-13 and IFNγ, which promote inflammation and exacerbate symptoms in IBD or allergic asthma models [84]. Exposure to conventional microbiota in early life decreases the CXCL16-mediated NK T cell recruitment, thereby preventing mucosal assemblage of NK T cells and ameliorating disease symptoms [85].

##### Innate Lymphoid Cells

Another example for innate immune cells with functional similarity to T cells are innate lymphoid cells (ILCs), which can be divided into the three main subgroups of ILC1 (T-bet^+^), ILC2 (GATA-binding protein 3^+^) and ILC3 (retinoic acid receptor-related orphan receptor-γt (RORγt)^+^) [21]. Some studies demonstrated a clear impact of the commensal microbiome on ILC (prominently ILC3) functionality such as increased secretion of IL-22 in germ-free mice. IL-22 is known to induce the secretion of anti-microbial peptides (e.g., lipocalin2, S100 and Reg3 proteins) by intestinal epithelial cells, which is required for defense against, e.g., *Citrobacter rodentium* infection of the intestinal mucosa [86]. Furthermore, the depletion of ILC3 in mice lead to dissemination of bacterial infection to the periphery and a concomitant systemic inflammation, which was attenuated by IL-22 administration [87]. Since the role of IL-22 is still controversially debated, additional studies are required to delineate its role in mucosal protection and immune regulation [88].

##### Mucosal-Associated Invariant T Cells

In previous years an additional subset of invariant cells comes to the fore as important players in inflammatory diseases [89]. The mucosal-associated invariant T (MAIT) cells, which are characterized by the expression of an invariant αTCR chain and the non-classical major histocompatibility complex-I related protein 1 [89,90,91]. This group of innate-like T cells is primarily located at mucosal tissues (e.g., intestinal lamina propria), and therefore plays a key role in the recognition of microbial and dietary antigens. Upon activation, MAIT cells produce diverse pro-inflammatory cytokines, such as IL-17, IFNγ, granzyme B or TNFα [89,90]. In vitro experiments with *Escherichia coli* and *Candida albicans* demonstrated that different activators evoke distinct cytokine expression profiles of MAIT cells [89,92]. This microbe-specific functional specialization enables the MAIT cells to precisely modulate and amplify inflammatory responses of other immune cells [89,90,92]. Due to their expression of various chemokine receptors, MAIT cells exhibit a migratory capacity into remote tissues [89,90]. In line with this, several publications describe the involvement of MAITs in autoimmune disease, such as IBD and MS, whereas the exact function and cell frequency in certain compartments is still controversially discussed [89,90,91,93].

#### 2.2.2. Adaptive Immune System

Besides the innate immune system, the adaptive immune system also critically interacts with the microbiome. The adaptive immune system tightly controls the composition of the intestinal microbiome by encouraging commensal bacteria and debilitating pathogenic microbes. Rag-deficient mice that lack B and T cells demonstrate an increase in microbiota richness and evenness, due to the absence of the controlling element [94,95]. In contrast, the microbiome also modulates the precise composition of effector CD4^+^ T cell subsets and also the balance between effector and regulatory CD4^+^ T cells in the GALT, which is important for an intact host immune system [96]. Diverse chronic inflammatory and autoimmune diseases are associated with a disturbance of this equilibrium in subset of effector CD4^+^ T cells [21]. For instance in IBD, chronic intestinal inflammation is characterized by an increase in frequency of pathogenic effector CD4^+^ T cells and a reduction in protective Treg cells, which in turn, results in pro-inflammatory systemic response [21].

##### Pro-Inflammatory Effector T Cells

The subsets of the T effector cells have distinct and contradictory activities. Hence a precise control is important for well-balanced immune responses [96]. The most prominent representatives of pro-inflammatory effector CD4^+^ T cells are T helper type 1 (Th1) and Th17 cells. Both cell subsets have been described to be affected by the gut microbiome [96], but special attention has been paid on Th17 cells. As related to ILC3, Th17 cells are prevalent in the intestine and are important for the gastro-intestinal host defense, since they secrete cytokines, which are involved in the regulation of inflammation (e.g., IL-17A, IL-17F and IL-22) [97]. In germ-free mice or antibiotic treated mice, the number of Th17 cells is reduced along with attenuated pro-inflammatory responses. Administration of segmented filamentous bacteria (SFB) improved development of Th17 cells in mice [98,99,100]. By attachment to intestinal epithelial cells, SFB provoke the secretion of acute phase proteins, such as serum amyloid A1 and A2, which favors Th17 cell differentiation [101,102]. Furthermore, IL-6, IL-23, transforming growth factor beta 1 (TGFβ1) and IL-1β are important for Th17 cell development [97]. Low-dose penicillin treatment of young mice attenuates IL-17 expression and, therefore, diminishes pathogen-induced Th17 cell differentiation in the lamina propria, which results e.g., in attenuated colitis caused by eradication of commensal SFB [103]. Very little is known about the interaction between the microbiota and Th1 cells. Interestingly, germ-free mice showed an impaired balance between Th1 and Th2 cells that could be restored via colonization with the PSA-producing *Bacteroides fragilis*. This further underlines the importance of the microbiota for an intact immune response [96].

##### Anti-Inflammatory T Regulatory Cells

The counterpart to the pro-inflammatory effector CD4^+^ T cells are anti-inflammatory regulatory T (Treg) cells, whose proportion is two to three-folds higher in the gastro-intestinal tract compared to other tissues [104]. Besides programming Th17 cells, the microbiome shapes Treg cell development and responses. Mice with a compromised gut microbiota, such as germ-free mice or after antibiotic treatment, display a reduced number of Treg cells as well as an impairment of their anti-inflammatory cytokine-secretion, especially of IL-10 [104,105,106]. Re-colonization of these mice with specific microbiota population, such as Clostridium species cluster IV and XIVa [104], the altered Schaedler flora [105], or the human commensal *Bacteroides fragilis* [74,107,108] increase the functionality and frequency of Treg cells. Their accumulation in the gastro-intestinal tract is also favored by microbial-derived SCFA via inhibition of histone deacetylation and diminished DNA-methylation in the Forkhead box P3 (FoxP3) locus, which drives Treg cell development [54,109,110,111]. Treg cells are important for the regulation of mucosal immune responses, e.g., by controlling expansion of effector T cells with specific T cell receptors against commensal bacteria. Thus, the administration of FOXP3-expressing Treg cells restores the decreased diversity in microbiota of T cell (Cd3e)-deficient mice without T cells [112]. This effect is mediated via the immune-modulating and anti-inflammatory function of Treg cells as well as their regulation of immunoglobulin A (IgA) selection in the germinal centers of the GALT (e.g., Peyer’s Patches), all being important mechanisms to maintain microbiome diversity and gastro-intestinal homeostasis [112]. This implicates a mutual interplay between Treg cells and the gastro-intestinal microbiota, which has been reviewed in detail by Tanoue et al. [113].

##### B Cells

The gastro-intestinal microbiome also influences B cells, which are another important component of the antigen-specific adaptive immune response. Bacterial antigens trigger differentiation, antigen presentation and antibody production of B cells either (a) directly via activation of receptors (e.g., TLR, B cell receptor) and via modulation of B cell metabolism [114,115], or (b) indirectly via induction of other immune cells [112]. Bacterial lipopolysaccharide (LPS) force class-switch recombination in B cells towards IgA production [115]. IgA is a dimeric immunoglobulin and produced in mucosal tissues, such as the intestine, at high concentrations. After receptor-mediated uptake of IgA by epithelial cells, IgA is secreted into the intestinal lumen, where it binds to bacterial or food antigens. This coating blocks the adhesion or the migration of potentially detrimental antigens and microbes into the lamina propria [115]. Beside supporting the intestinal barrier function, IgA shapes the microbiome composition and thereby also functionality [116,117]. In particular, disturbances of activation-induced cytidine deaminase (AID) or co-receptor programmed cell death 1 (PD-1) are identified to be responsible for alterations in the microbiome composition [116,117,118].

In conclusion, the gut–CNS-axis represents a promising target to modulate chronic inflammatory processes via supporting intrinsic protective immune-regulatory mechanisms.

## 3. The Role of the Gut–CNS-Axis in Multiple Sclerosis

In MS various pre-clinical and clinical studies indicated that the gut–CNS-axis exhibits the potential to enforce protective processes and to attenuate pathological mechanisms in the context of CNS autoimmunity.

### 3.1. Pre-Clinical Studies

Pre-clinical studies of MS have deployed different murine models of EAE. Beside mice with spontaneously developing EAE symptoms, there are also inducible EAE-models, where mice are actively immunized with a myelin peptide antigen combined with complete Freud’s adjuvant and pertussis toxin (actively induced EAE) [119,120]. In several of those experimental models a clear link between the gastro-intestinal microbiota composition and pathological effects in the CNS has been demonstrated. Mice treated with antibiotics or maintained under germ-free conditions have a compromised gastro-intestinal microbiome. Under these circumstances disease onset in spontaneous EAE mice was attenuated [119,121], and mice with actively induced EAE displayed a significant decrease in disease severity [120,122,123,124]. In line with other inflammatory diseases, there is a disturbed balance in immune responses, particularly in T cell responses, in MS/EAE. The pathology of MS/EAE is characterized by an enhancement of pro-inflammatory effector T cells and a decrease in CD4^+^CD25^+^FoxP3^+^ Treg cell frequency and an impaired Treg function [2,3]. Notably, the protective effect observed in germ-free or antibiotic treated mice with EAE is associated with a correction of this imbalance in T cell responses in the gastro-intestinal tract and the CNS. Specifically, in the intestine as well as in the CNS pro-inflammatory cells, such as Th1 and Th17 cells or their cytokines IFNγ and IL-17A, are diminished, whereas the extent of the anti-inflammatory T reg cell response, including secretion of IL-10 and IL-13, are increased [119,120,121,122,123,124,125,126]. Beyond alterations in T cell subsets, recruitment of B cells and the ability of dendritic cells to activate Th1 and Th17 cells was impaired in microbiota-compromised mice in diverse EAE-models [119,120]. On the other hand, colonization of microbiota-compromised mice with SFB that enhance Th17 cells facilitated EAE disease susceptibility [119,120].

In summary, the gut microbiome is crucially involved in the modulation of neuroinflammation in EAE. Hence, it has been postulated that targeted alteration of the gut microbiome might, in turn, promote protective intrinsic mechanisms. Indeed, administration of PSA-producing *Bacteroides fragilis* or a probiotic mixture of three *Lactobacilli* strains raised numbers of Treg cells and regulatory B cells to attenuate EAE symptoms [75,76,77,125]. As mentioned previously, treatment with probiotic *Lactobacillus farciminis* decreased intestinal permeability [57], which has been postulated to be associated with murine EAE [127] plus MS [128,129]. A reduction of the intestinal hyperpermeability resulted in attenuated HPA-axis responses and neuroinflammatory activity [57]. Hence, promotion of protective versus pathogenic effects mediated by the gut–CNS-axis depend on specific microbiota constellations; however, further investigations are required to understand the complex interactions and to select, e.g., the moist effective pre- and probiotics.

### 3.2. Clinical Studies

So far, a translation of these preclinical results to human MS is not simple. First indications about a role of the gut–CNS-axis with regard to disease susceptibility of MS patients, but also of MS-related NMO-patients, can be derived from several recent cross-sectional case-control studies [32,130,131,132,133,134,135]. Overall, significant differences between the microbiome composition of healthy controls and MS patients have been reported concerning the relative richness and diversity of the gastro-intestinal microbiota at the taxa level. However, these changes of the microbiota composition were rather mild [130,131,132,133,134,135]. In two case-control studies *Methanobrevibacter* and *Akkermansia* were enriched in MS-Patients [131,133]. These intestinal mucosa-associated microbes are important for host homeostasis and contribute to inflammation [131,133,136]. *Methanobrevibacter smithii* was shown to activate dendritic cells and recruit inflammatory cells, in part via an adjuvant effect of its lipids [133]. The role of the mucosa-degrading *Akkermansia muciniphila* in inflammatory processes is controversially discussed. On the one hand, degradation of the mucosa provokes the collapse of the intestinal barrier integrity, which results in increased levels of inflammatory MAMPs in the circulation potentially contributing to neuroinflammation [133,136]. On the other hand, the absence of dietary glycans *Akkermansia muciniphila* is also known to ferment host-derived mucins into SCFA, which exhibits neuroprotective functions [133]. Thus, further investigation is required, to clarify the circumstances of the protective versus possibly also inflammatory action of *Akkermansia muciniphila* interference. In contrast, a reduction in *Bacteroidetes* and *Firmicutes* was unanimously described in MS patients [131,132,134,137]. Some representatives of *Bacteroidetes* are known to improve host defense, whereas others are related to pathological processes. Depending on the context, *Bacteroides fragilis* can induce anti-inflammatory mechanisms as mentioned previously, although it often acts as clinical pathogen [138,139]. Furthermore, acetate-utilizing butyrate producing members of Clostridia clusters XIVa and IV were also diminished in two case-control studies with MS patients [130,140]. These microbes have been described to contribute to various chronic inflammatory diseases, including IBD [141] or celiac diseases [142]. *Faecalibacterium prausnitzii* belongs to the Clostridia species and exhibits anti-inflammatory properties mediated by butyrate production as well as secretion of microbial anti-inflammatory molecules [141,143]. In this protective context, *Faecalibacterium prausnitzii* reduces intestinal permeability in inflammatory diseases [144].

Due to the heterogeneity and often insufficient stratification of the studies in MS patients only few consistent results have been obtained so far. Other bacterial genera, such as *Prevotella* or *Dorea*, were also shown to be significantly regulated in MS patients, but contradicting results were obtained. In order to comprehend the entire complexity of the gut–CNS-axis involvement in MS, further investigations with well-defined patient cohorts are required, to evidence the preliminary indications in humans and strengthen the significance of effects described in animal models.

## 4. Conclusions and Further Perspectives

Our review illustrates the relevance of the gut–CNS-axis as a promising target for therapeutic interventions in MS. There is a clear interaction and complex regulation between the intestinal microbiota, the intestinal immune system and the ENS as well as CNS. These interactions finally shape and modulate neuronal functionality as well as systemic and even local CNS inflammatory processes. Such targeted modification of the gut–CNS-axis e.g., by dietary interventions and/or specific probiotics represents a promising complementary approach in addition to conventional immune therapies in MS. Subsequently, this will likely increase treatment success without concomitant increase in treatment-related severe side effects, such as general immune suppression or occurrence of secondary autoimmune diseases, which are a well-recognized problem of highly-active drugs for MS. However, further studies are urgently needed to better understand nutritional influences on the gut microbiome and the gut–CNS-axis. To achieve this, well performed proof-of-concept studies followed by large multi-center double-blind randomized studies are required, with a strict control of dietary habits and standard guidelines for collection and processing of fecal samples. Moreover, the potential disturbance of gut microbiome composition and activities by MS drugs themselves has been taken into consideration, since antimicrobial properties of Dimetylfumarate, Fingolimod and Teriflunomide have recently been uncovered [137]. Beside their robust anti-oxidative or anti-inflammatory effects, these drugs might also act via alteration of the gut microbiome profile [137]. Thus, closer examination of the microbiota composition in MS patients in the context of specific immune therapies is warranted. The ultimate goal is to delineate a clear picture of beneficial versus detrimental dietary habits (and resultant microbiota), to provide precise dietary guidelines as a complementary treatment approach in MS.

## Abbreviations

AID	Activation-induced cytidine deaminase
ANS	Autonomic nerve system
CD	Cluster of differentiation
CNS	Central nervous system
CXCL16	Chemokine (C-X-C motif) ligand 16
EAE	Experimental autoimmune encephalomyelitis
ENS	Enteric nervous system
FoxP3	Forkhead box P3
GABA	Gamma-aminobutyric acid
GALT	Gut-associated lymphatic tissue
HPA axis	Hypothalamic–pituitary–adrenal axis
IBD	Inflammatory bowel disease
IFN	Interferon
IgA	Immunoglobulin A
IL	Interleukin
ILC	Innate immune lymphoid cell
MAMP	Microbial-associated molecular pattern
MS	Multiple sclerosis
NK cell	Natural killer cell
NMO	Neuromyelitis optica
PD-1	Programmed cell death 1
PSA	Polysaccharide A
RORγt	Retinoic acid receptor-related orphan receptor-γt
SCFA	Short-chain fatty acids
SFB	Segmented filamentous bacteria
TGFβ1	Transforming growth factor β1
Th1 cell	T helper cell type 1
Th17 cell	T helper cell type 17
TLR	Toll-like receptors
TNFα	Tumor necrosis factor α
Treg cell	Regulatory T cell
